# Supplementary figures and images for: Chronic IL-1 exposure drives LNCaP cells to evolve androgen and AR independence
Source: PLoS One. 2020 Dec 16;15(12):e0242970. doi: 10.1371/journal.pone.0242970 (PMC7743957; doi:10.1371/journal.pone.0242970)

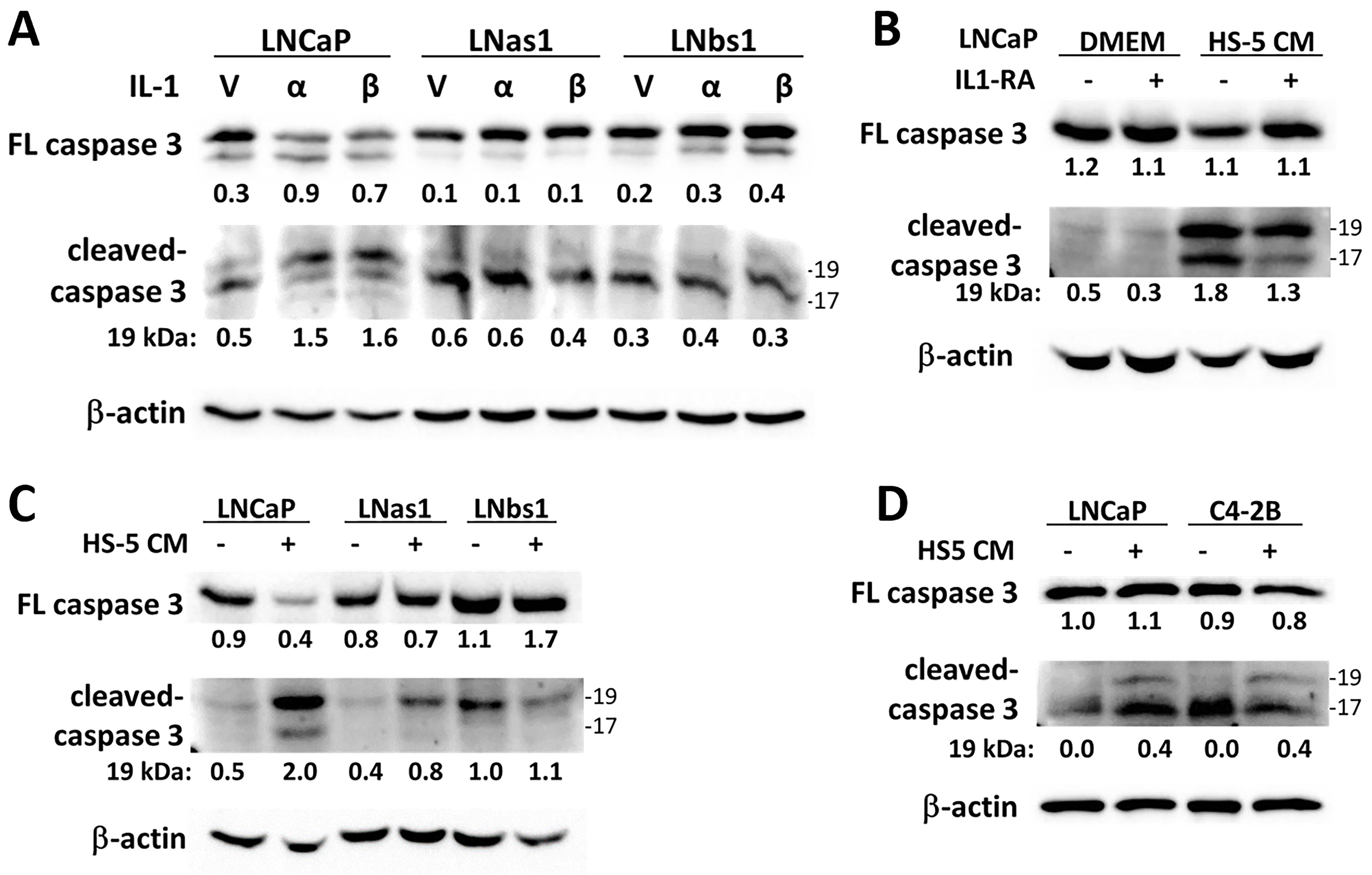

Supplement: S1 Fig — (A) LNCaP, LNas1 and LNbs1 cells were treated for 3 days with vehicle control or 25 ng/ml IL-1α or IL-1β and analyzed for full-length (FL) caspase 3 protein accumulation using a caspase 3 antibody or caspase 3 low molecular weight cleavages products using a cleaved caspase 3 antibody. Detection of full-length caspase 3 turnover or cleavage products indicates activation of apoptosis. IL-1 induced cleavage of full-length caspase 3 and induced the accumulation of the 19 KDa caspase 3 cleavage product in LNCaP, but not LNas1 or LNbs1 cells. (B) LNCaP cells were pre‐treated for 1 day with vehicle control or 400 ng/ml human recombinant IL‐1RA and the following day the medium was replaced with treatment control (DMEM) or HS‐5 conditioned medium (CM) plus an additional 400 ng/ml IL‐1RA or vehicle control for 3 additional days. HS-5 CM reduced full-length caspase 3 and induced the accumulation of the 19 KDa and 17 KDa caspase 3 cleavage products. IL-1RA attenuated accumulation of the caspase 3 cleavage products. (C, D) LNCaP, LNas1, LNbs1 and C4-2B cells were treated for 3 days with treatment control or HS-5 CM. HS-5 CM reduced the accumulation of full-length caspase 3 and/or induced the accumulation of the 19 KDa and/or 17 KDa cleavage products in LNCaP and C4-2B cells, but not in LNas1 or LNbs1. Taken together, LNas1 and LNbs1 have reduced sensitivity to IL-1-induced apoptosis activation. Full-length caspase 3 cleavage densitometry shows the ratio of cleaved to uncleaved caspase 3. Low molecular weight 19 KDa and 17 KDa caspase 3 cleavage product densitometry is normalized to β-actin. (TIF) [file pone.0242970.s002.tif]

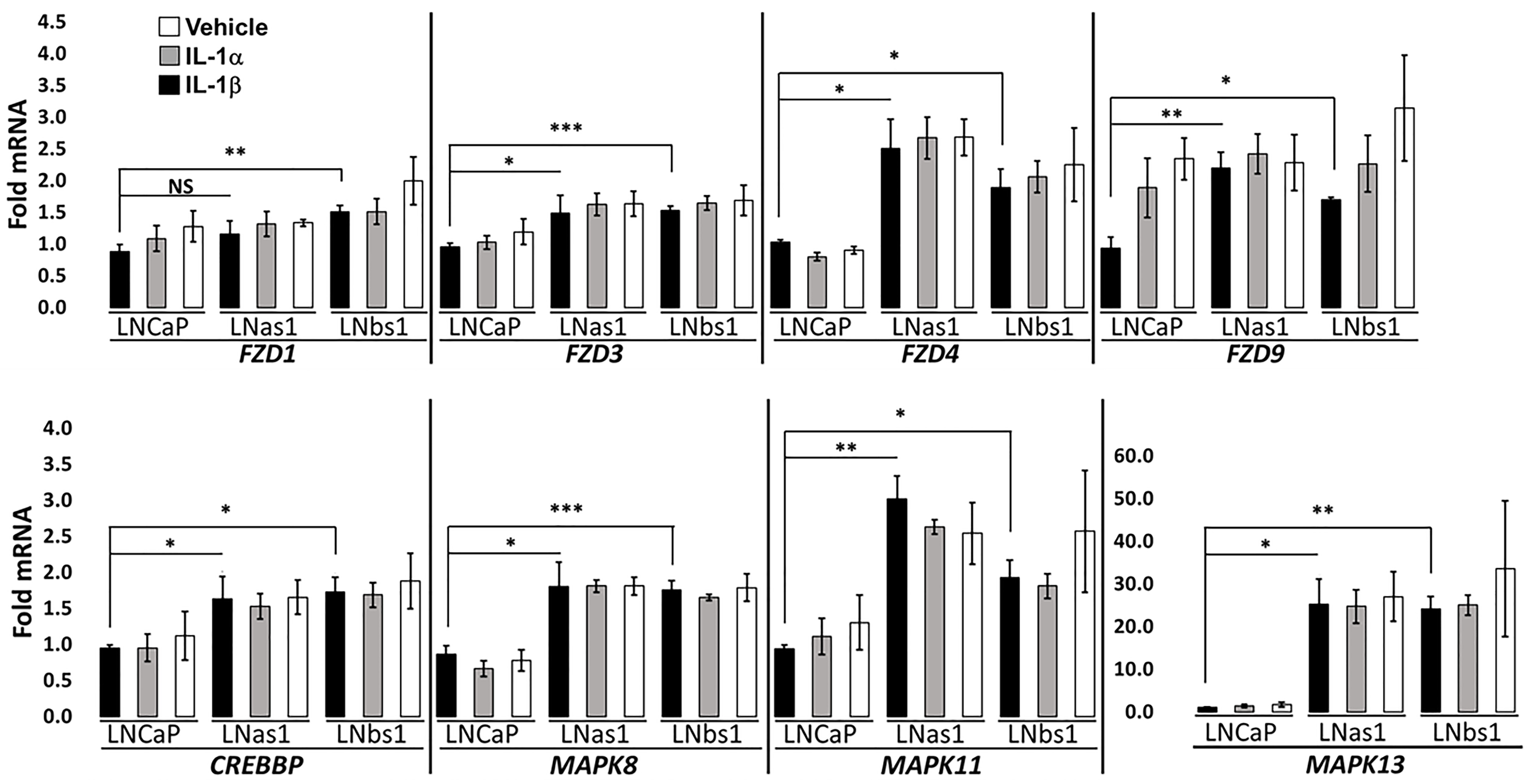

Supplement: S2 Fig — LNCaP, LNas1 and LNbs1 cells were treated for 3 days with vehicle control or 25 ng/ml IL-1α or IL-1β and analyzed for mRNA levels by RT-qPCR for FDZ1, FDZ3, FDZ4, FDZ9, CREBBP, MAPK8, MAPK11, MAPK13. These genes were chosen arbitrarily from S1 Table IPA analysis to represent a cross section of the EGF, AMPK, Wnt/Ca2+, NGF, FGF and ILK pathways. While acute IL-1 exposure did not modulate the expression level of the genes in LNCaP, LNas1 or LNbs1, basal gene expression was high in LNas1 and LNbs1. Error bars, ± STDEV of 3 biological replicates; p-value, *≤0.05, **≤0.005, ***≤0.0005, NS = not significant. Fold mRNA levels are normalized to LNCaP vehicle control for IL-1 treatments in order to also compare basal levels between the cell lines. (TIF) [file pone.0242970.s003.tif]

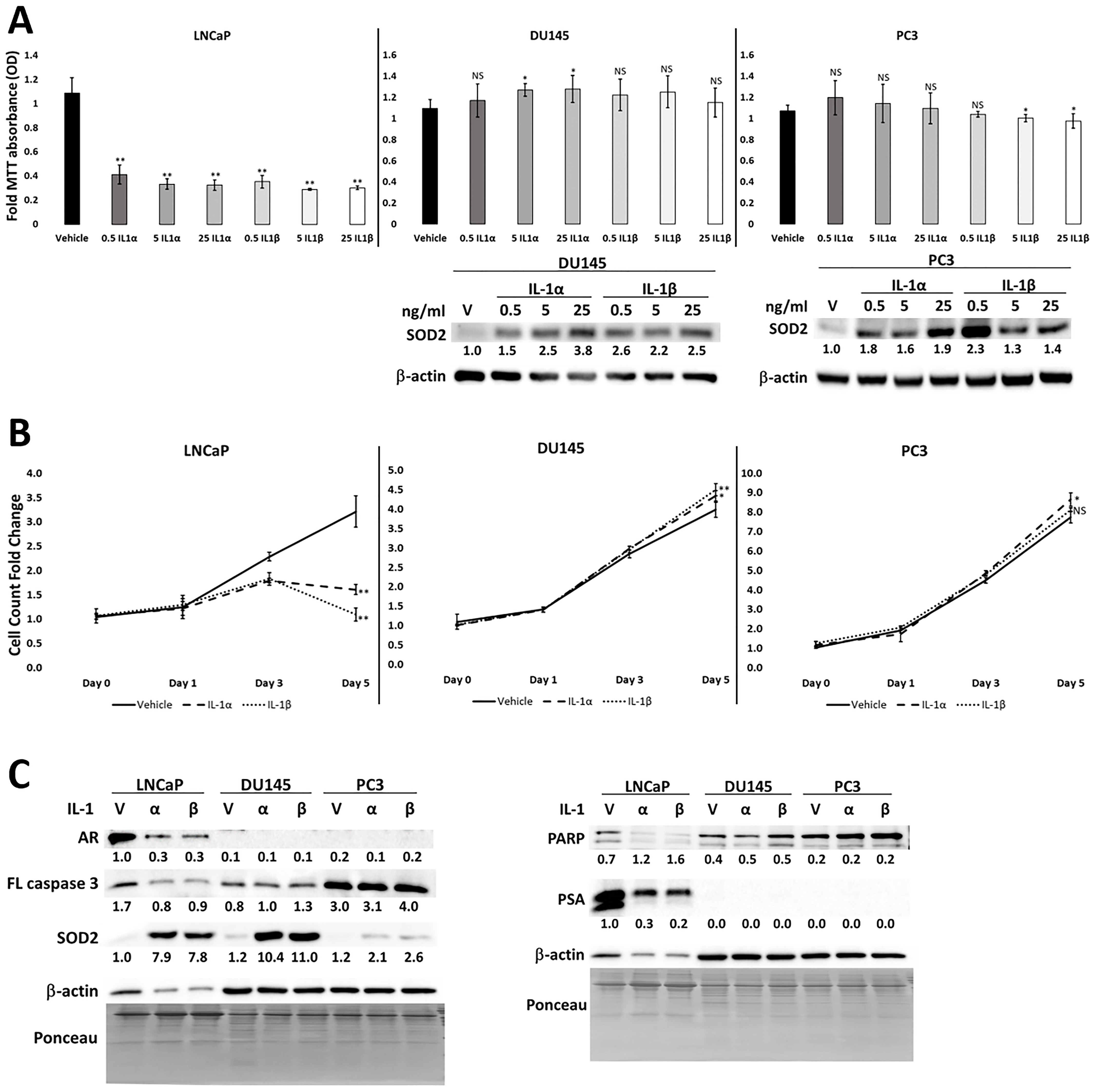

Supplement: S3 Fig — (A) LNCaP, DU145 and PC3 cells were treated for 3 days with vehicle control or 0.5–25 ng/ml IL-1α or IL-1β and analyzed for cell viability using MTT or for IL-1-induced intracellular signaling using SOD2 protein accumulation. LNCaP cells are sensitive to IL-1 induced cytotoxicity. No cells remained for protein analysis. DU145 and PC3 cells activated IL-1 intracellular signaling, but were not sensitive to IL-1-induced cytotoxicity. (B) LNCaP, DU145 and PC3 cells were treated for 0–5 days with vehicle control or 25 ng/ml IL-1α or IL-1β and viable cell counts determined. IL-1 reduced LNCaP cell number over time, but had no appreciable effect on DU145 or PC3 cell counts. (C) LNCaP, DU145 and PC3 cells were treated for 5 days with vehicle control or 25 ng/ml IL-1α or IL-1β and analyzed for AR, PSA, SOD2, PARP cleavage, and full-length caspase 3. As expected, IL-1 reduced AR, PSA and full-length caspase 3 accumulation and induced SOD2 accumulation and PARP cleavage in LNCaP cells. AR-negative DU145 and PC3 cell lines showed IL-1-induced SOD2 accumulation, but did not show IL-1-induced caspase 3 turnover or PARP cleavage. Error bars, ± STDEV of 3 biological replicates; p-value, *≤0.05, **≤0.005, NS = not significant. Fold MTT optical density (OD) and cell counts are normalized to the treatment control. Low β-actin in IL-1-treated LNCaP cells reflects cell death; therefore, protein bands for LNCaP, DU145 and PC3 were normalized to ponceau for densitometry and PARP densitometry shows the ratio of cleaved to uncleaved PARP. (TIF) [file pone.0242970.s004.tif]
