# Supplementary material for: Chronic IL-1 exposure drives LNCaP cells to evolve androgen and AR independence
Source: PLoS One. 2020 Dec 16;15(12):e0242970. doi: 10.1371/journal.pone.0242970 (PMC7743957; doi:10.1371/journal.pone.0242970)
Supplement: S1 Raw images — (PDF) [file pone.0242970.s005.pdf]

FIGURE 1B:

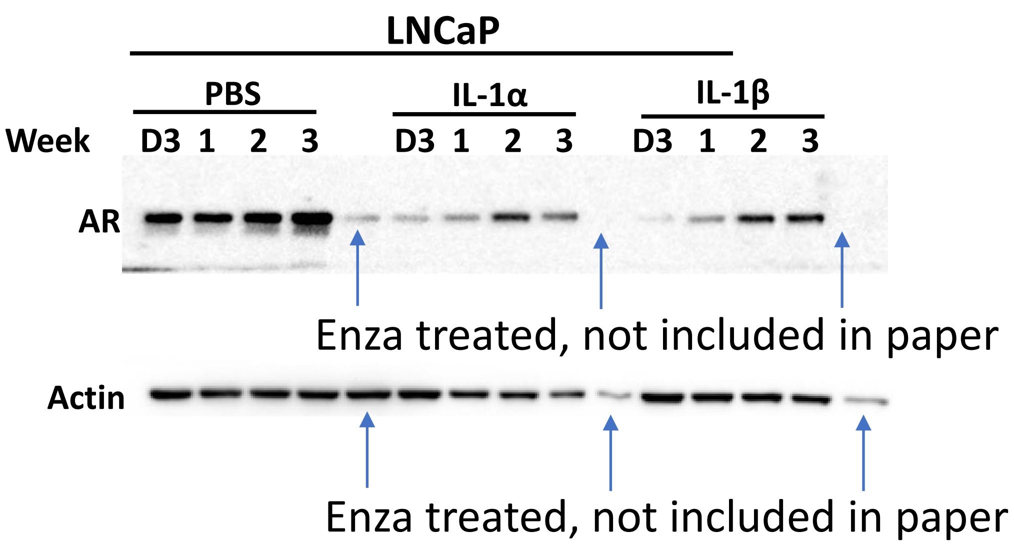

LNCaP
PBS
IL-1α
IL-1β
Week D3 1 2 3 D3 1 2 3 D3 1 2 3
AR
Enza treated, not included in paper
Actin
Enza treated, not included in paper

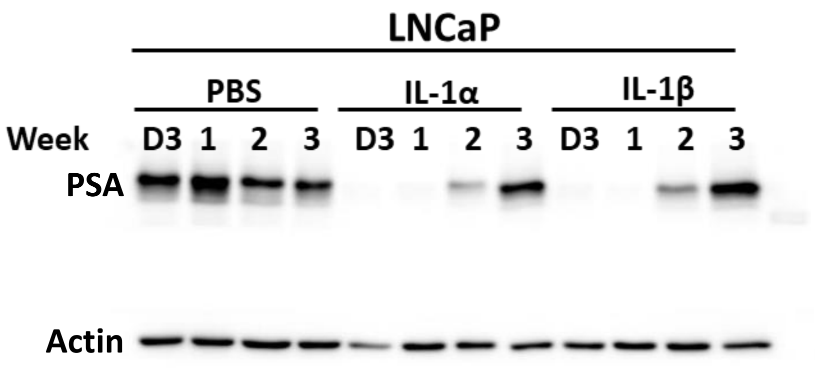

LNCaP
PBS
IL-1α
IL-1β
Week D3 1 2 3 D3 1 2 3 D3 1 2 3
PSA
Actin

FIGURE 2B:

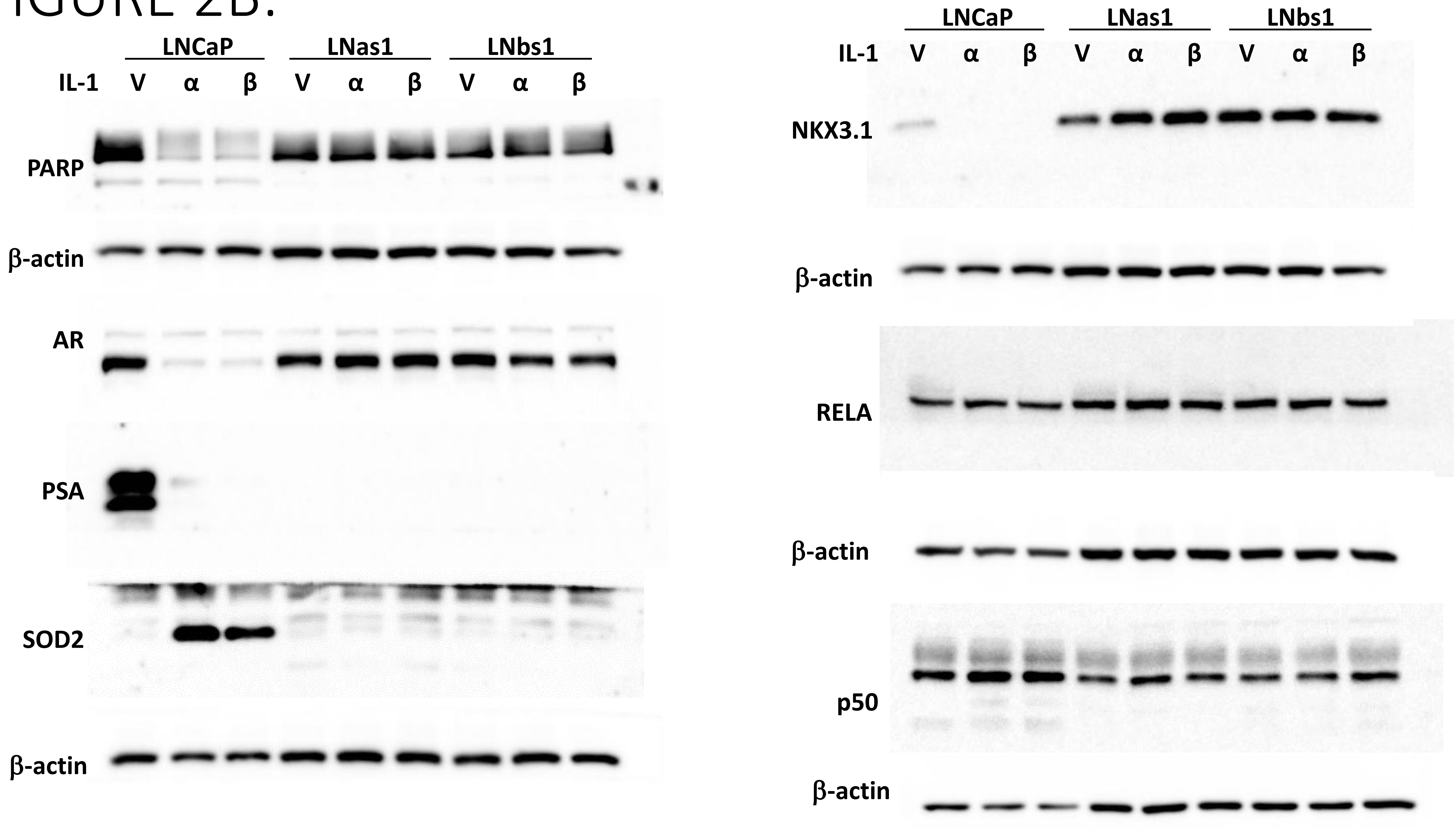
LNCaP
LNas1
LNbs1
IL-1
V
α
β
V
α
β
V
α
β
PARP
β-actin
AR
PSA
SOD2
β-actin
NKX3.1
β-actin
RELA
β-actin
p50
β-actin

FIGURE 3A:

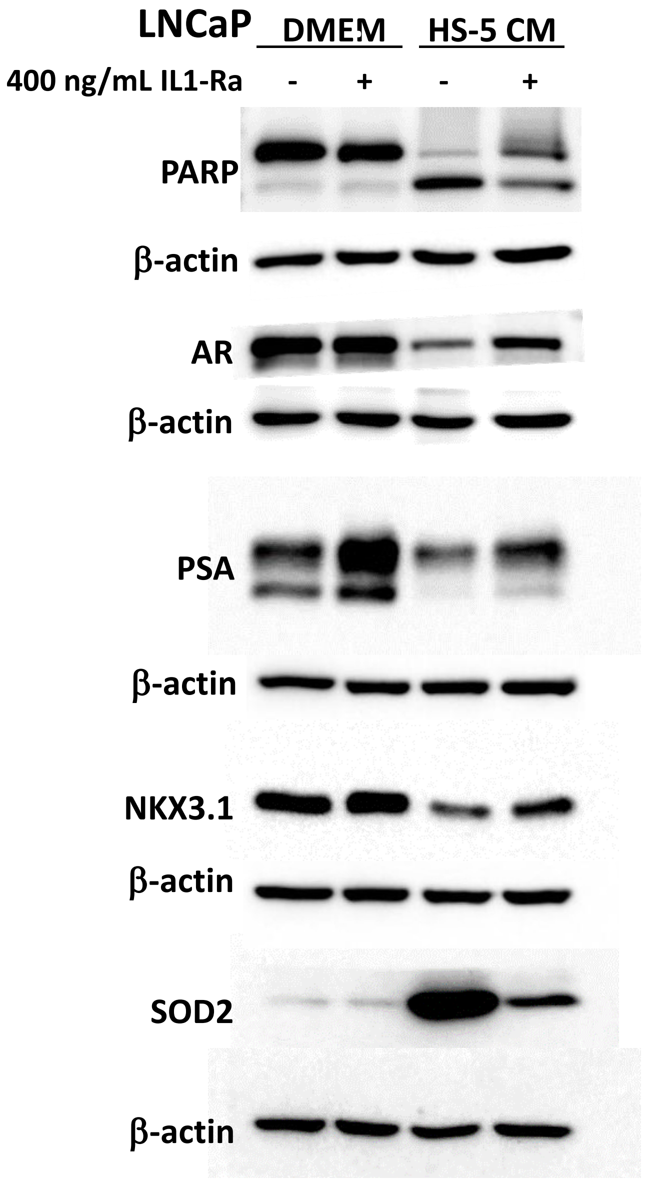

LNCaP
DMEM
HS-5 CM
400 ng/mL IL1-Ra
-
+
-
+
PARP
β-actin
AR
β-actin
PSA
β-actin
NKX3.1
β-actin
SOD2
β-actin

FIGURE 3C:

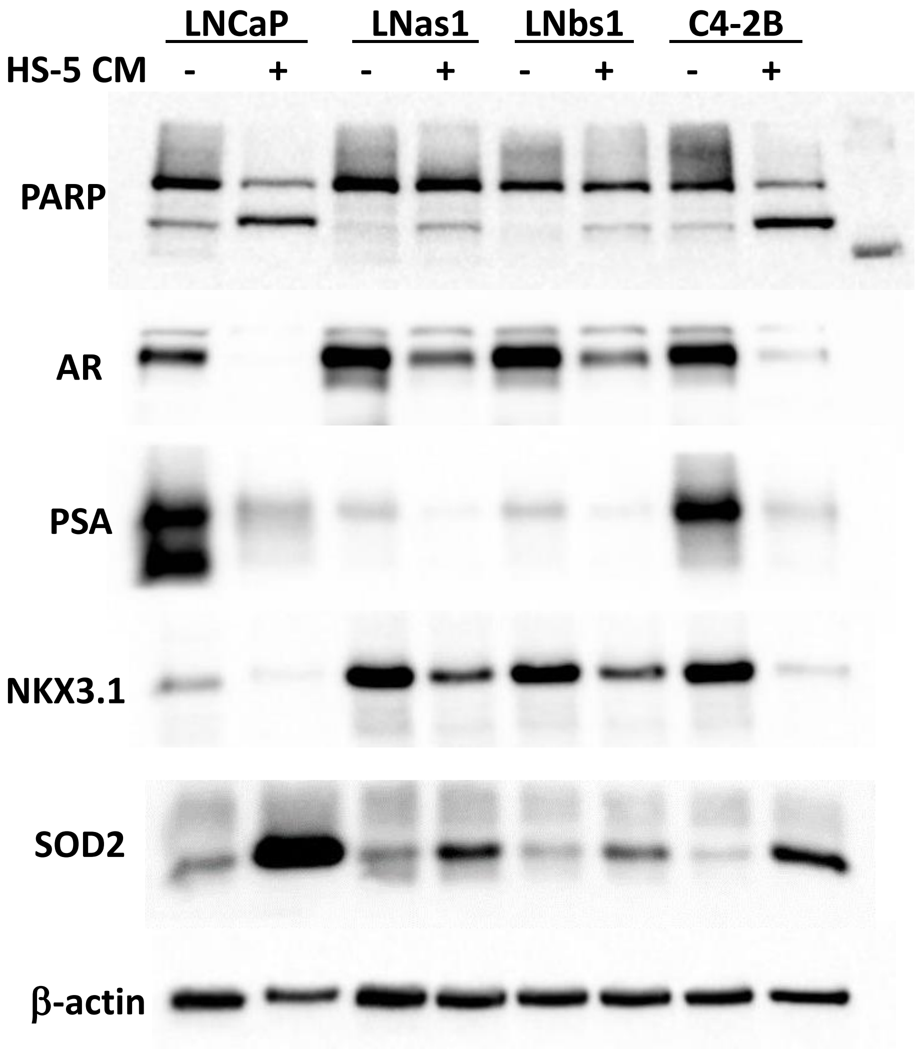

FIGURE 4D:

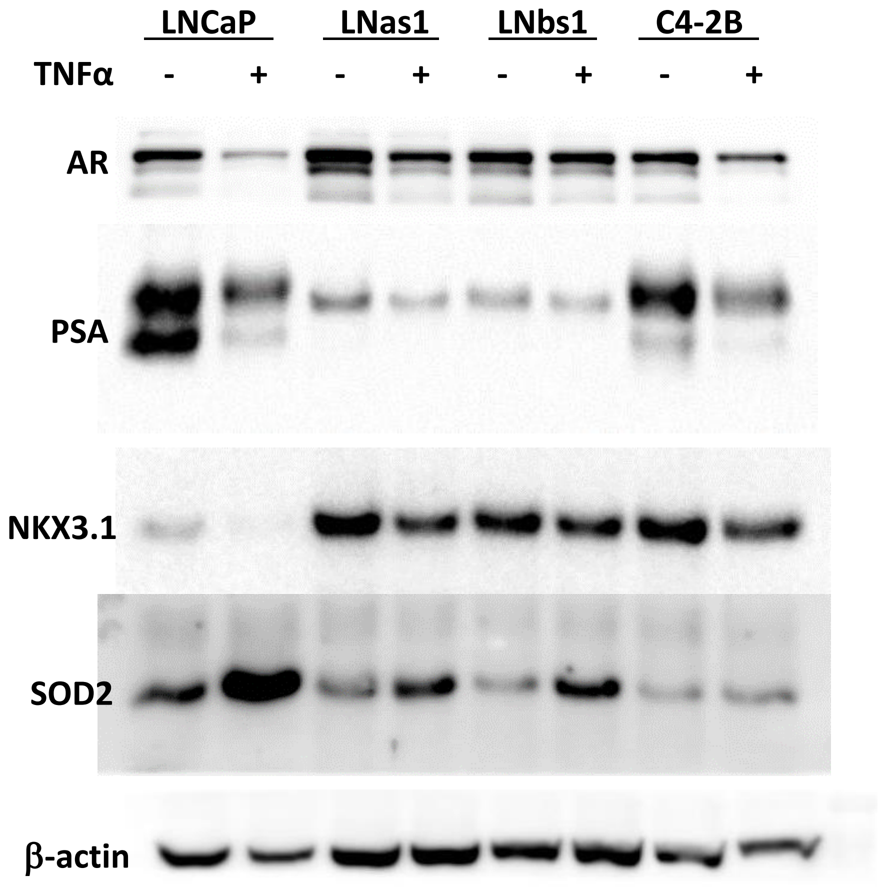

FIGURE 5C:

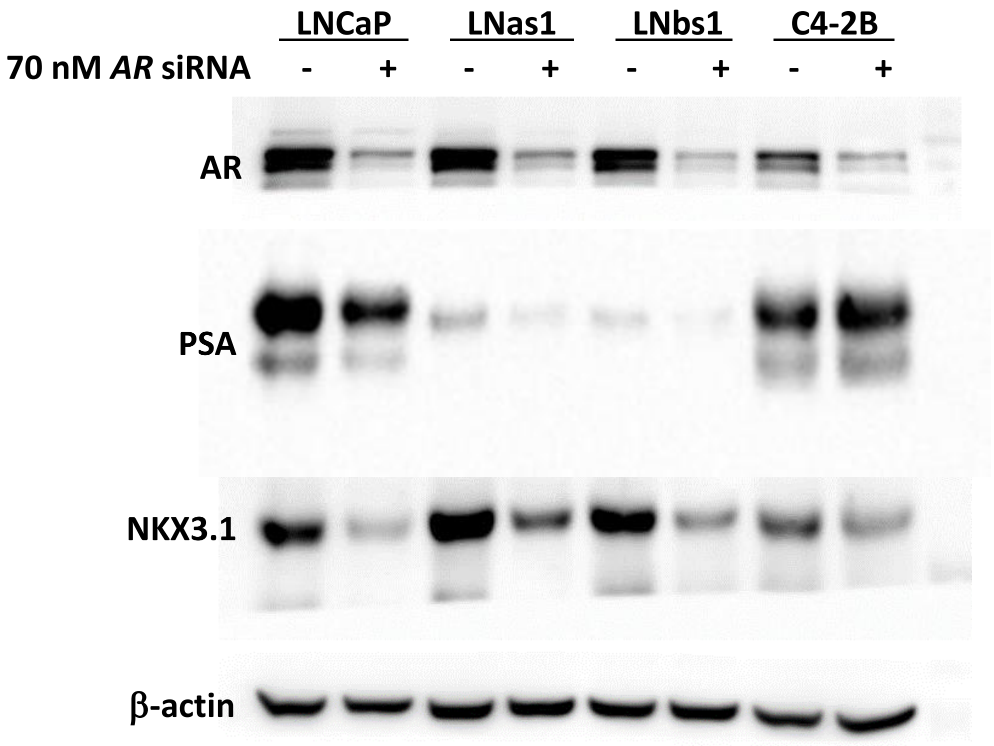

FIGURE 5D:

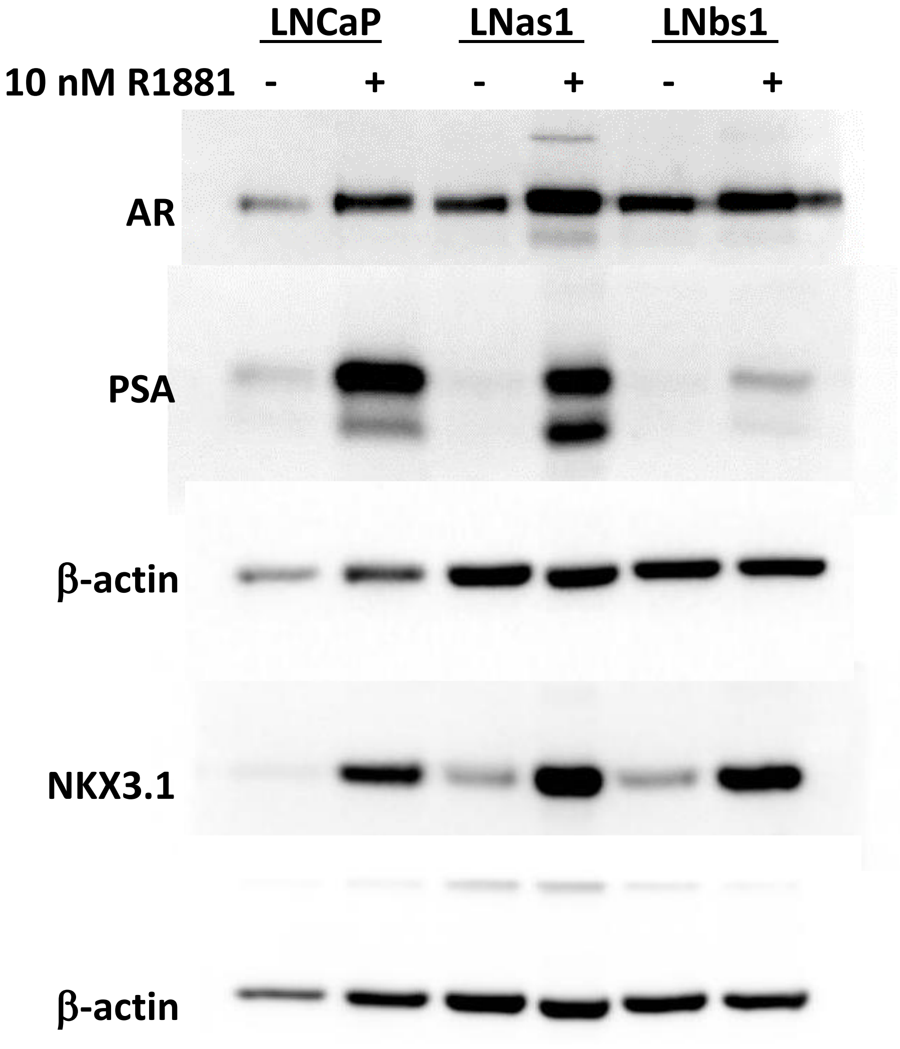

FIGURE 5E:

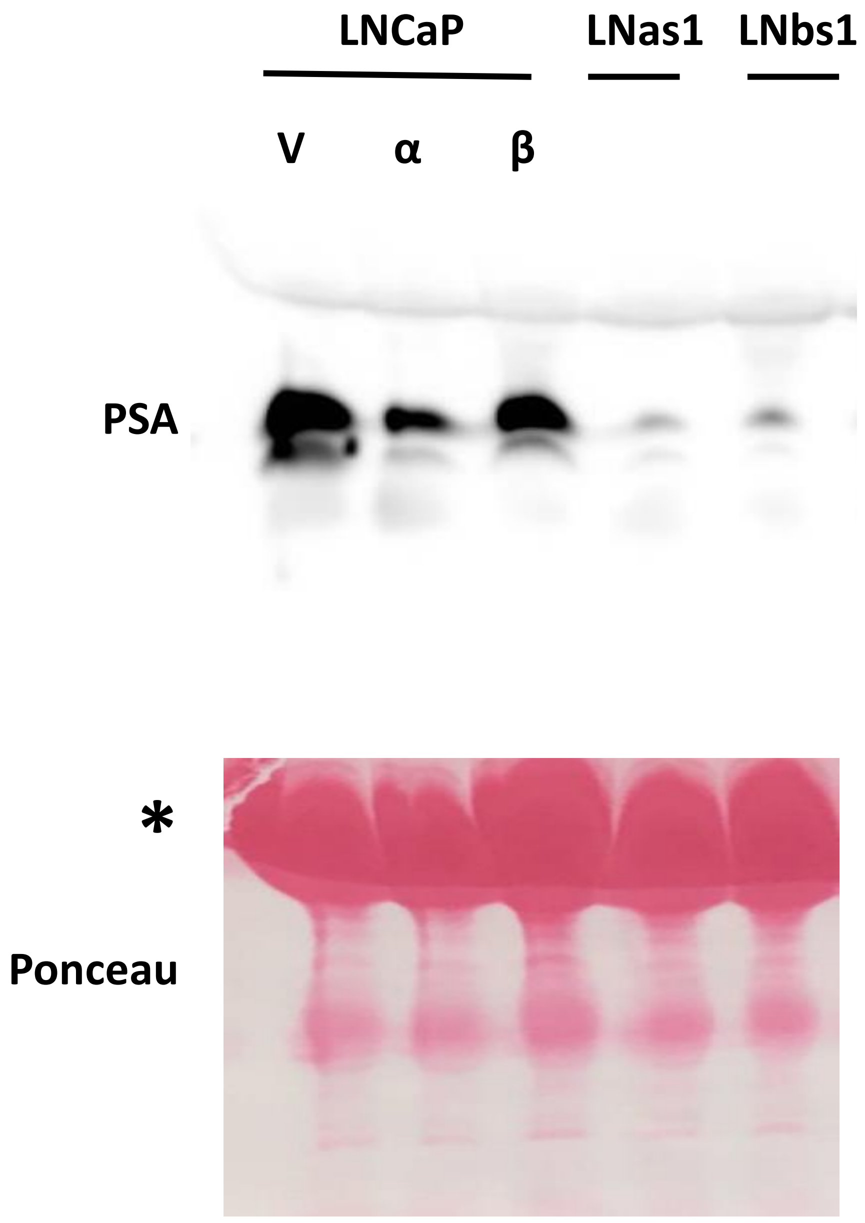

FIGURE 5F:

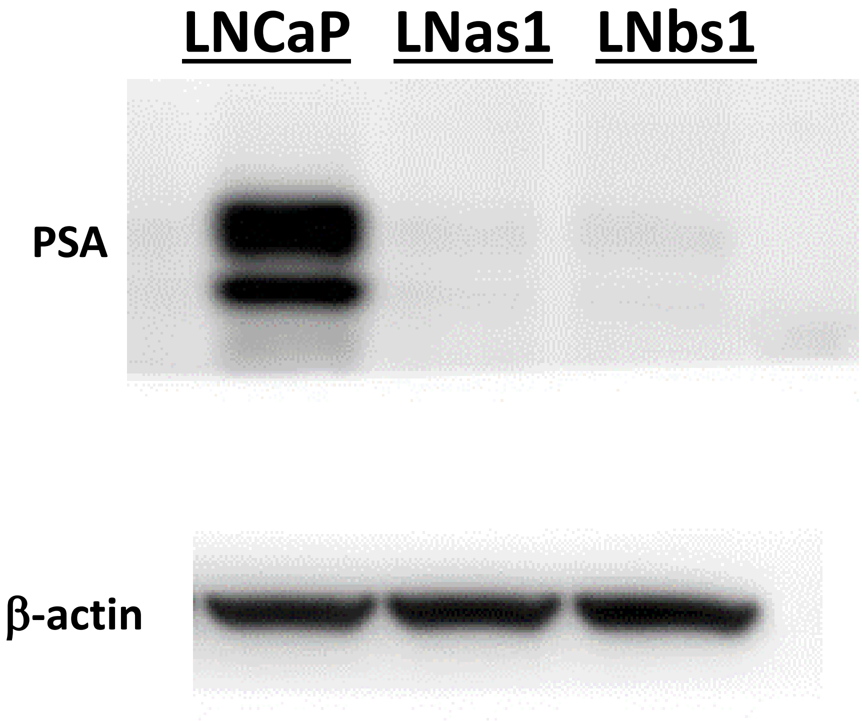

FIGURE 6B:

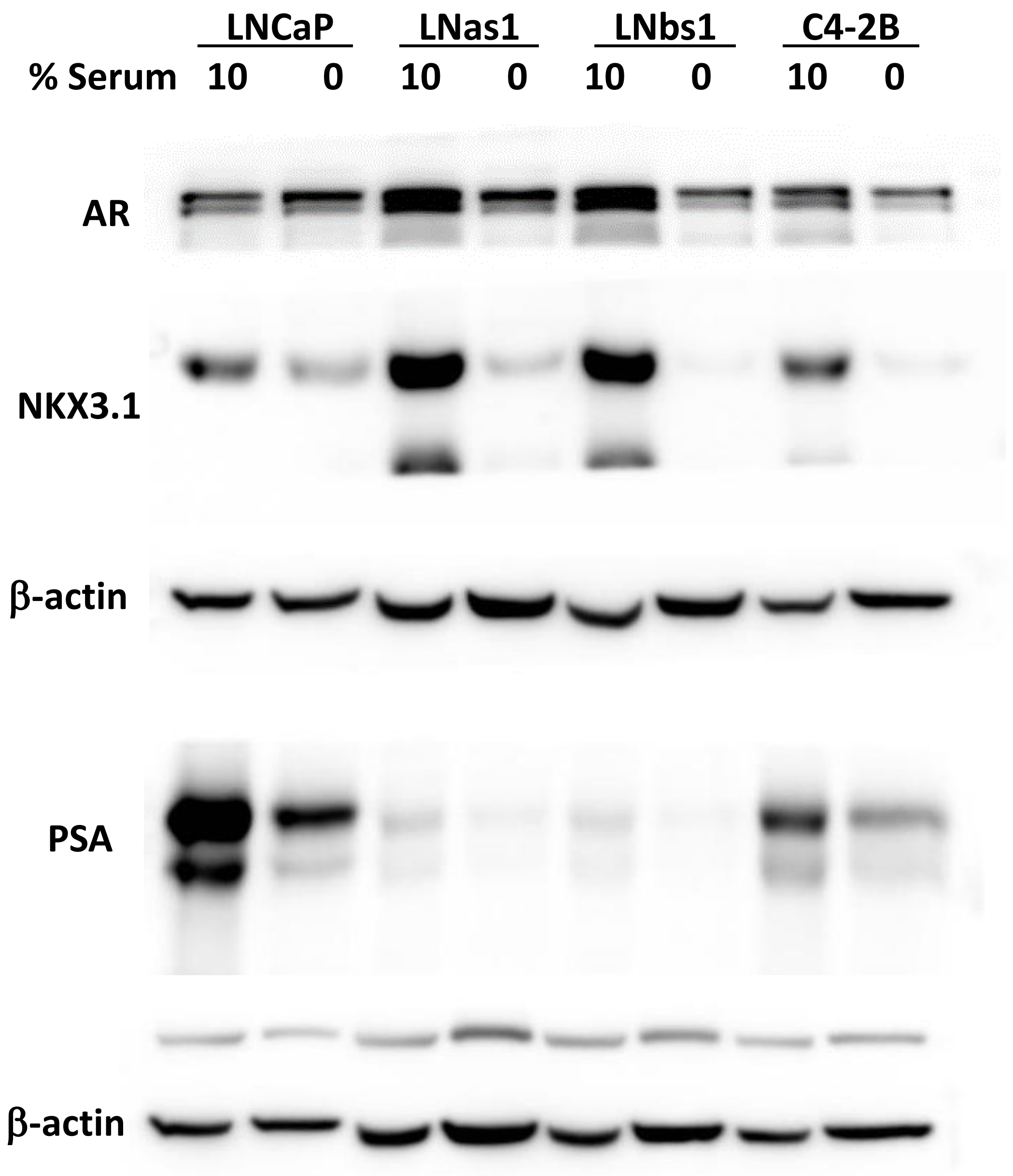
LNCaP
LNas1
LNbs1
C4-2B
% Serum 10 0 10 0 10 0 10 0
AR
NKX3.1
β-actin
PSA
β-actin

FIGURE 6C:

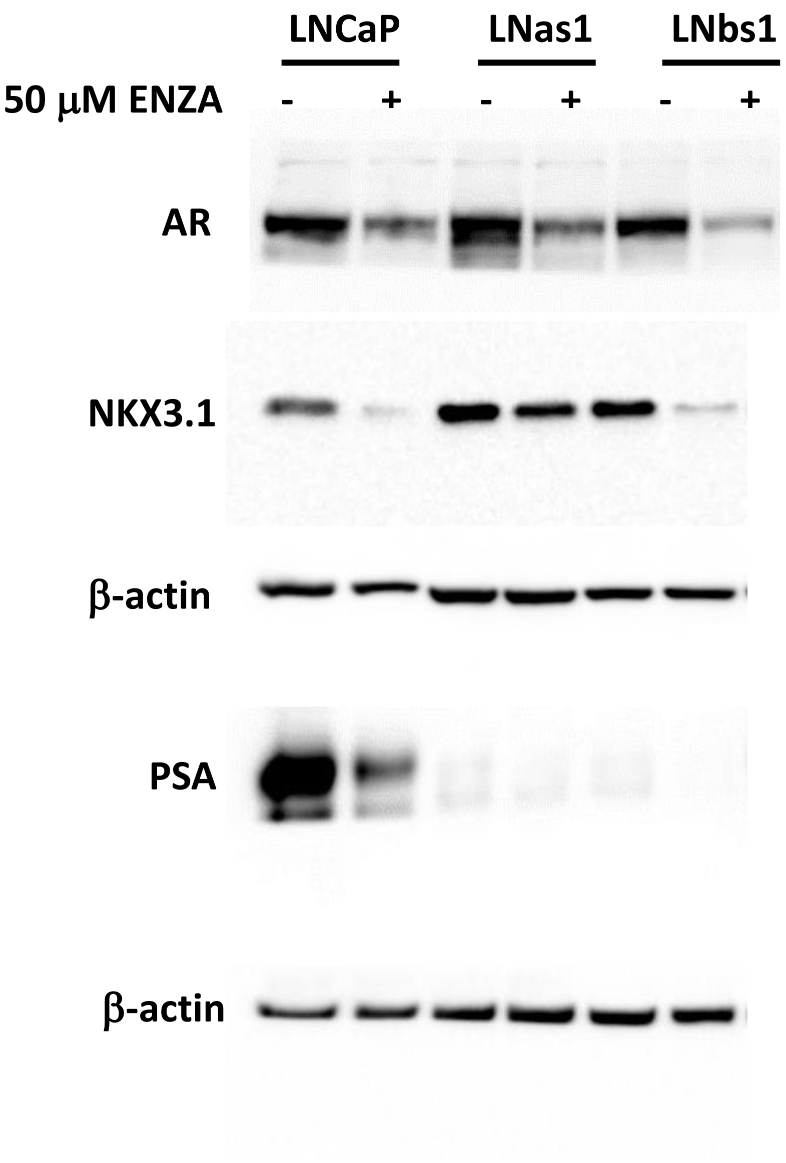

SUPP FIG 1A:

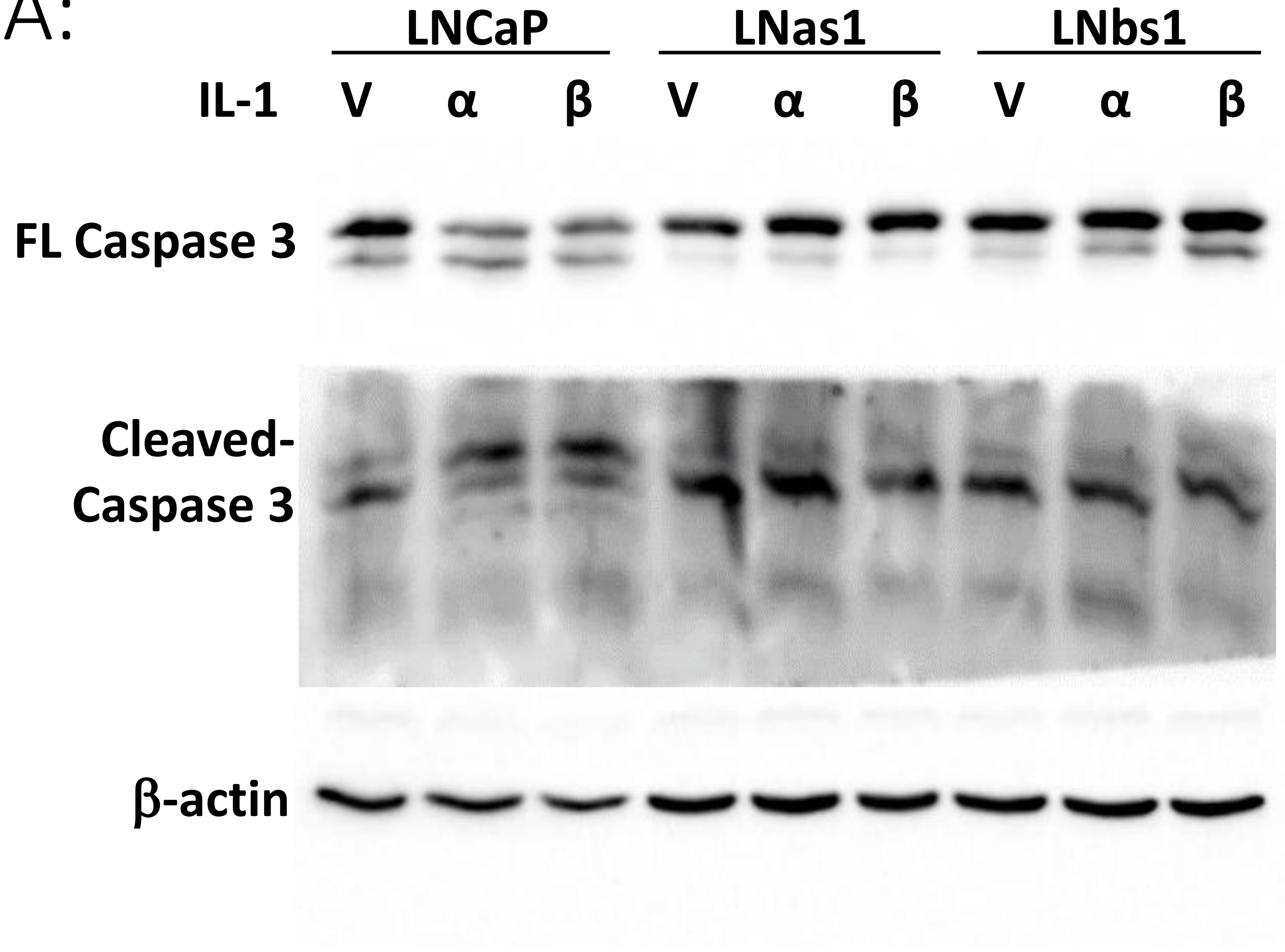
LNCaP
LNas1
LNbs1
IL-1
V α β V α β V α β
FL Caspase 3
Cleaved-
Caspase 3
β-actin

SUPP FIG 1B:

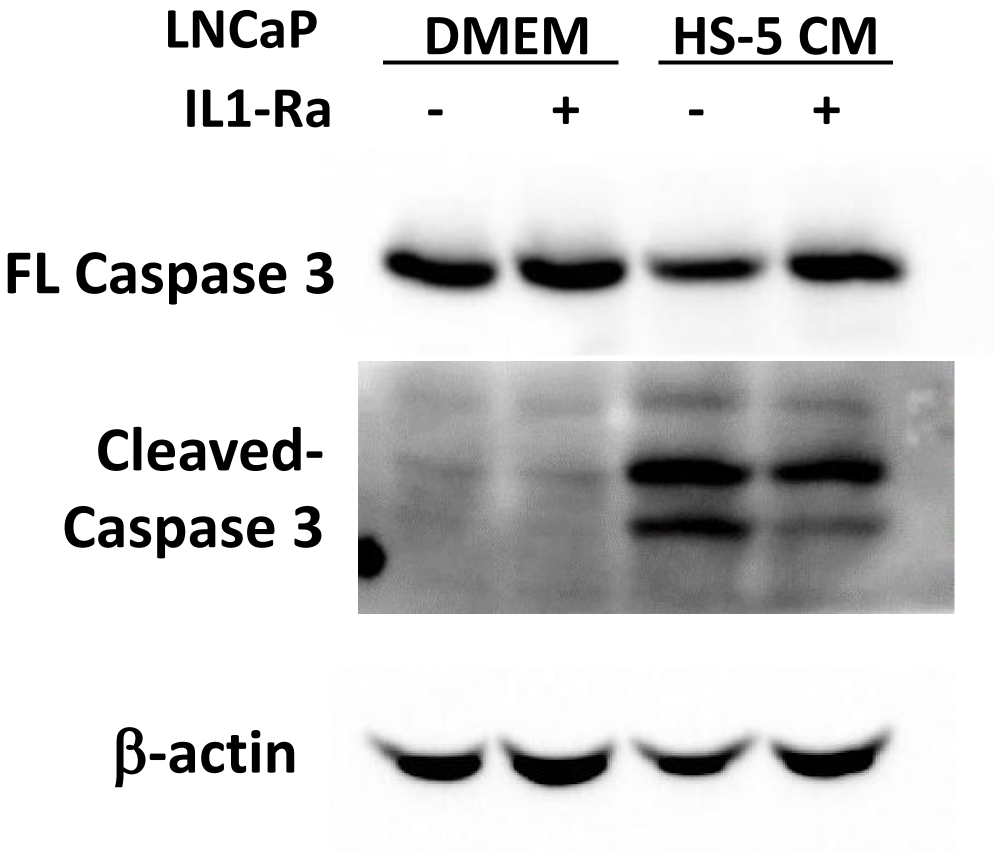
LNCaP
DMEM
HS-5 CM
IL1-Ra
-
+
-
+
FL Caspase 3
Cleaved-
Caspase 3
β-actin

SUPP FIG 1C:

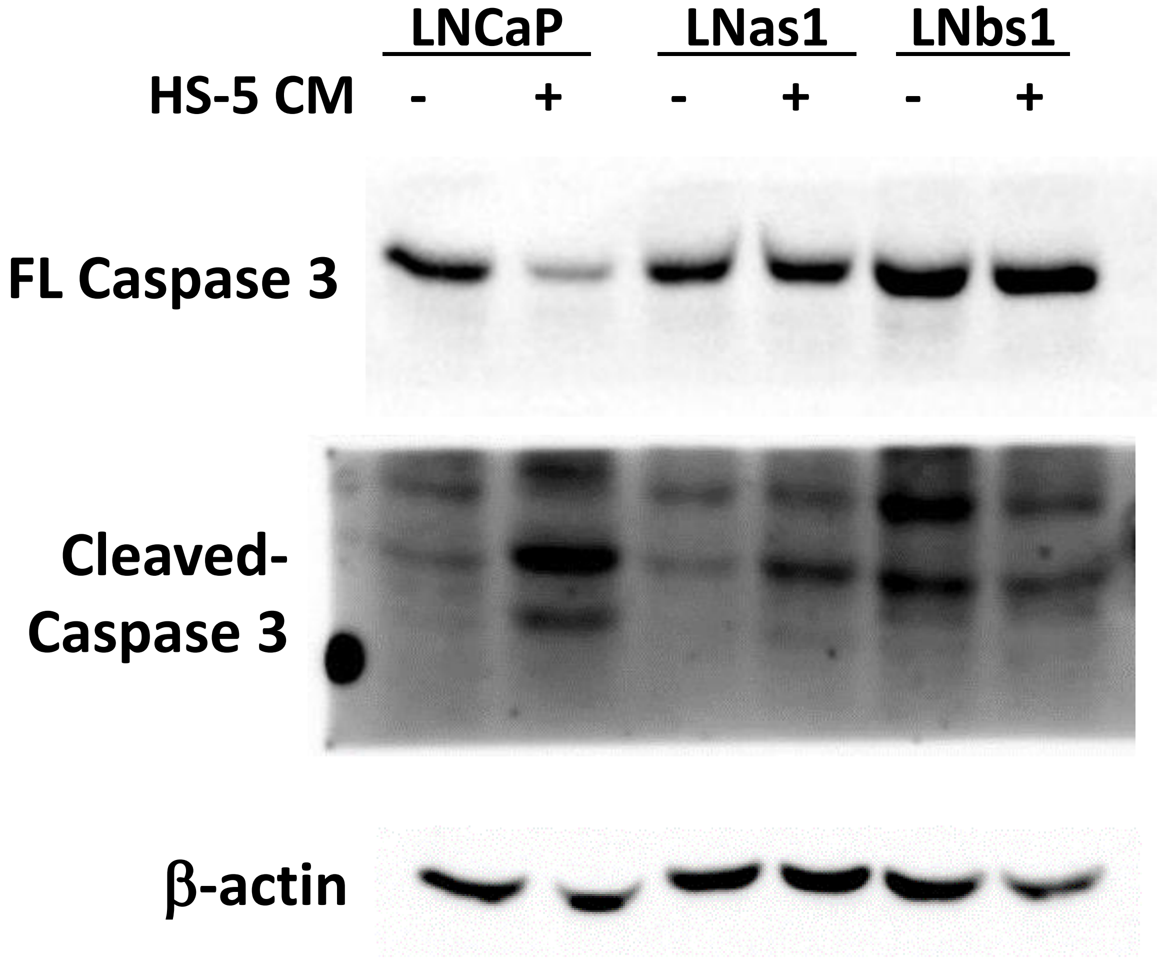
LNCaP
LNas1
LNbs1
HS-5 CM
-
+
-
+
-
+
FL Caspase 3
Cleaved-
Caspase 3
β-actin

SUPP FIG 1D:

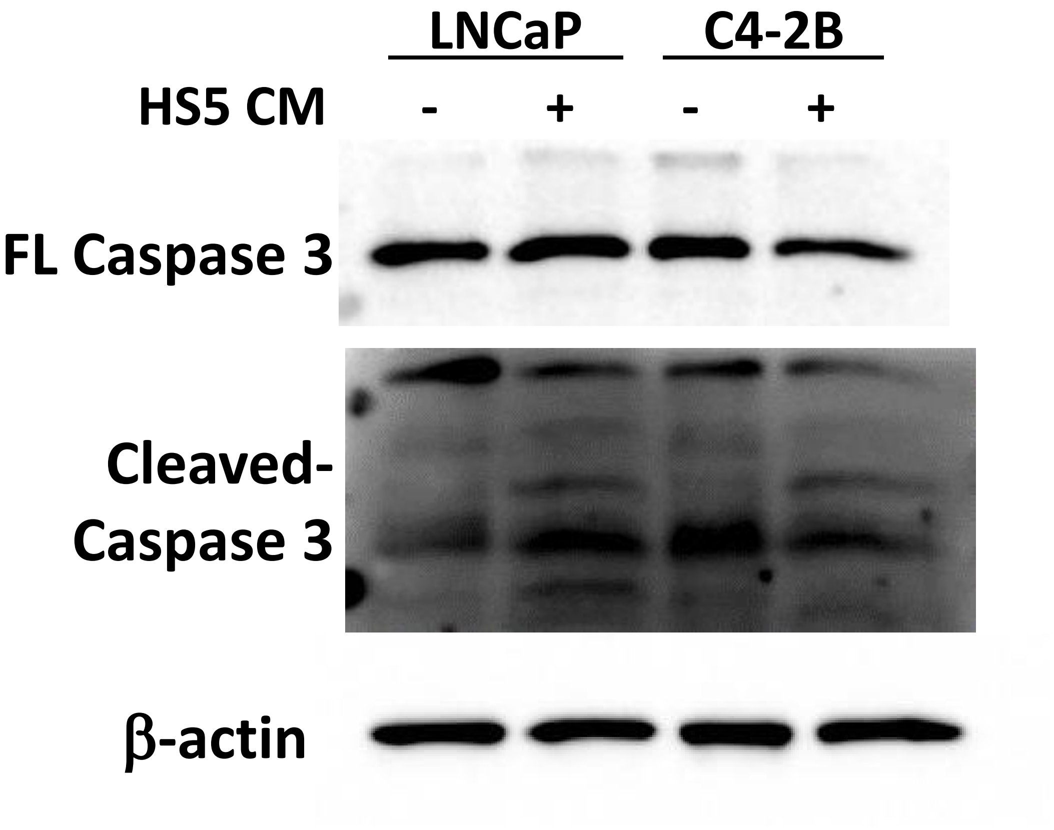

SUPP FIG 3A:

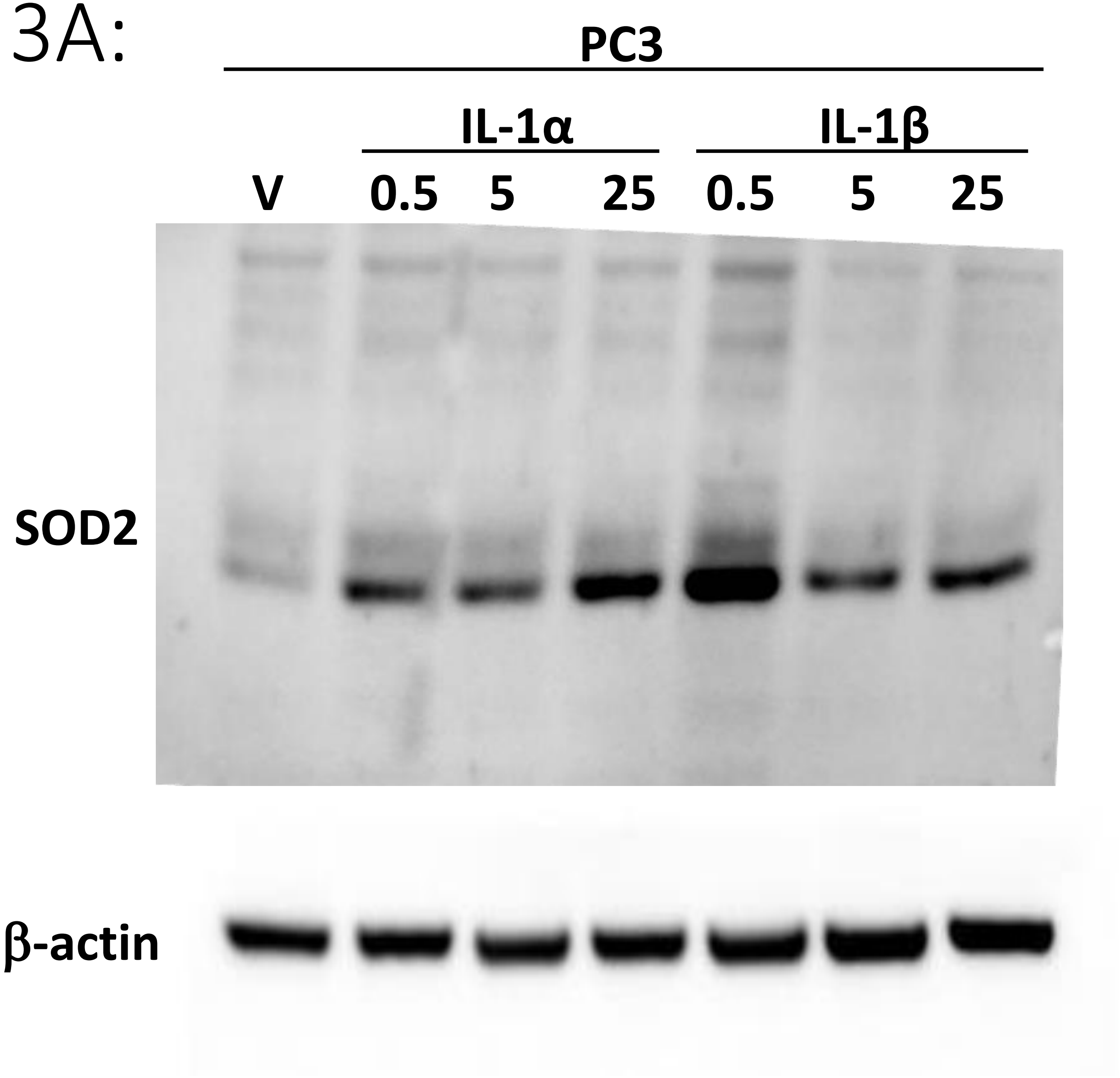

SUPP FIG 3A:

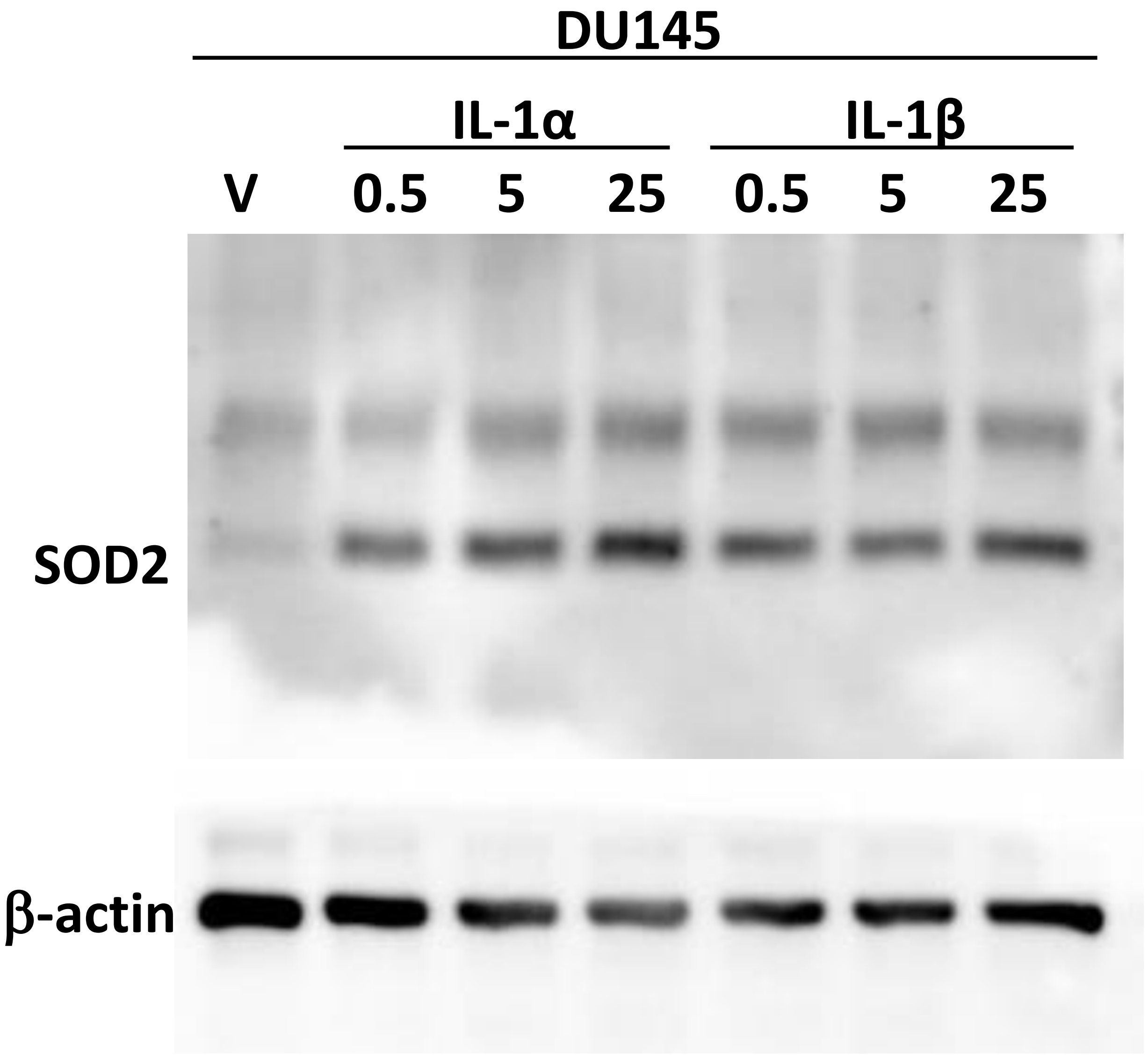

SUPP FIG 3C:

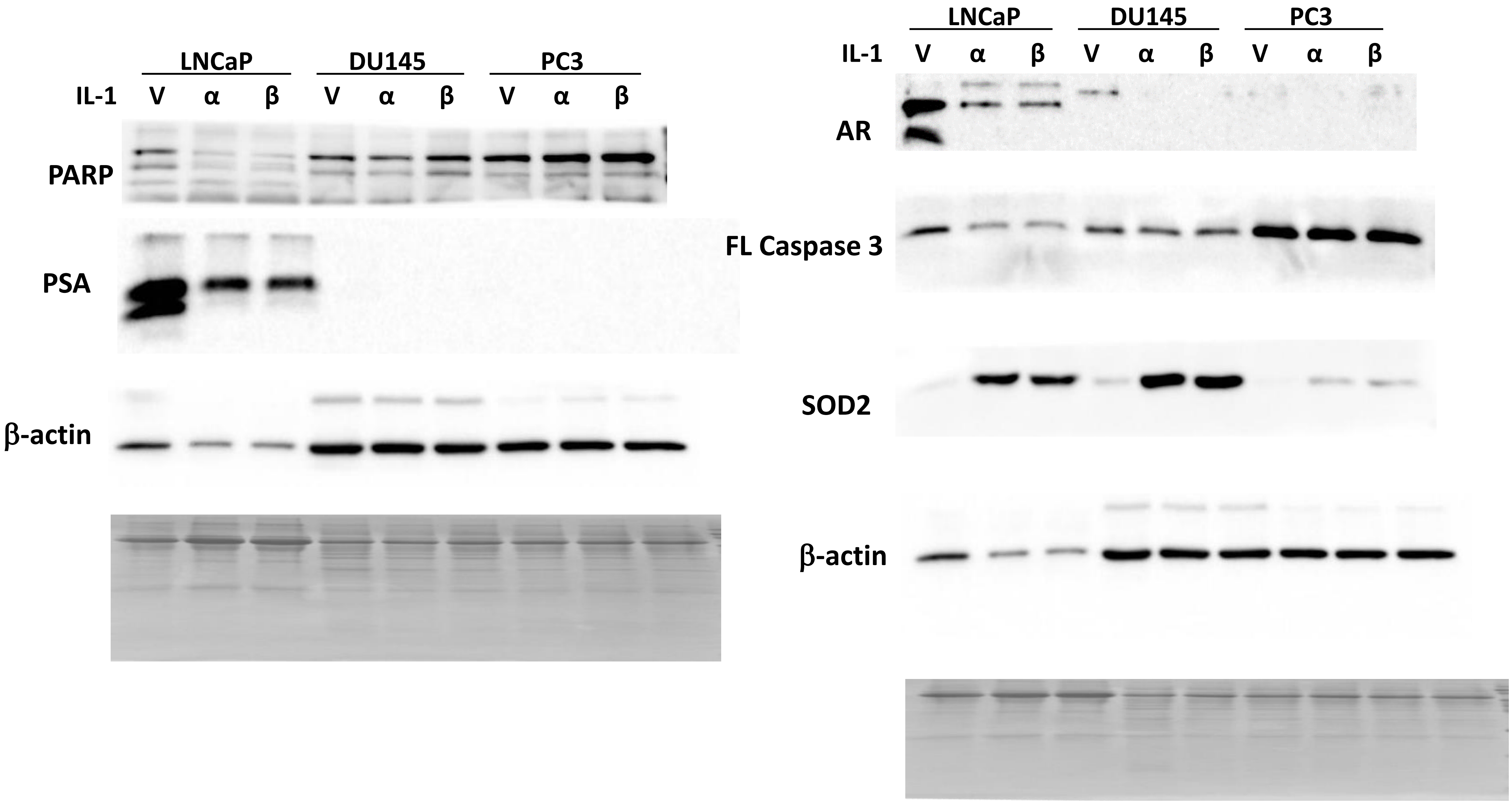
LNCaP
DU145
PC3
IL-1
V
α
β
V
α
β
V
α
β
PARP
PSA
β-actin
LNCaP
DU145
PC3
IL-1
V
α
β
V
α
β
V
α
β
AR
FL Caspase 3
SOD2
β-actin
